# Nonlocal Spin Valves Based on Graphene/Fe_3_GeTe_2_ van der Waals Heterostructures

**DOI:** 10.1021/acsami.2c21918

**Published:** 2023-02-08

**Authors:** Xin He, Chenhui Zhang, Dongxing Zheng, Peng Li, John Q. Xiao, Xixiang Zhang

**Affiliations:** † Physical Science and Engineering Division, King Abdullah University of Science and Technology (KAUST), Thuwal 23955-6900, Saudi Arabia; ‡ State Key Laboratory of Electronic Thin Film and Integrated Devices, University of Electronic Science and Technology of China, Chengdu 610054, China; § Department of Physics and Astronomy, University of Delaware, Newark, Delaware 19716, United States

**Keywords:** Fe_3_GeTe_2_, graphene, van der Waals heterostructure, nonlocal spin valve, spin transport

## Abstract

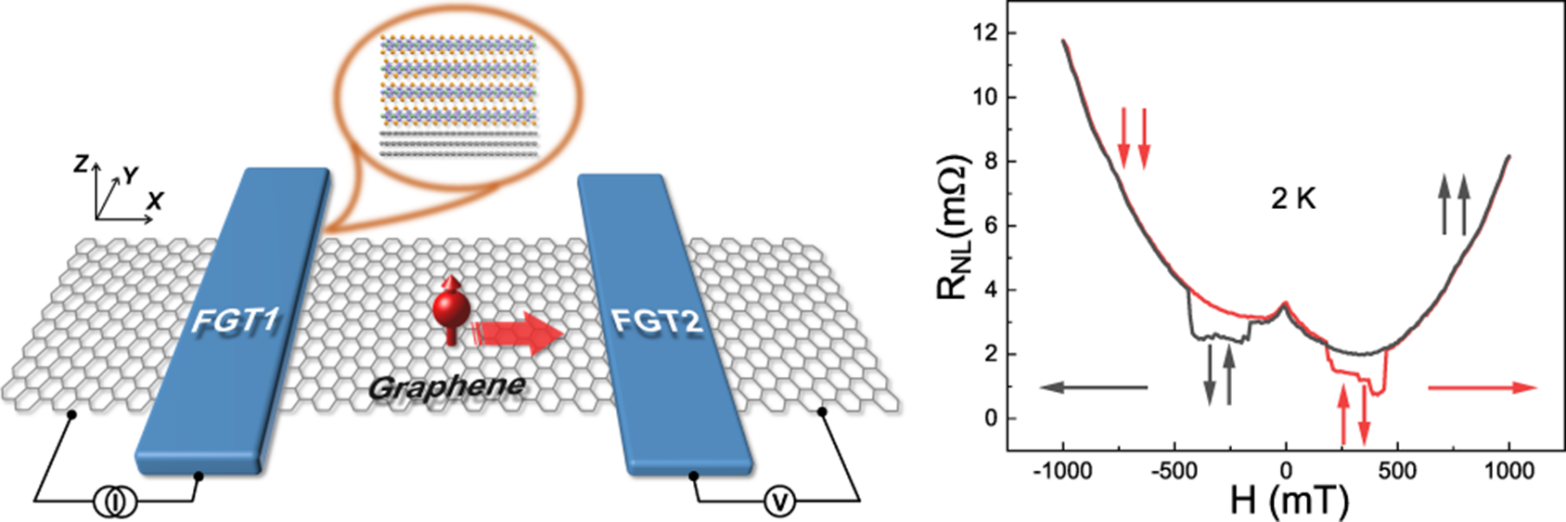

With recent advances in two-dimensional (2D) ferromagnets with enhanced Curie temperatures, it is possible to develop all-2D spintronic devices with high-quality interfaces using 2D ferromagnets. In this study, we have successfully fabricated nonlocal spin valves with Fe_3_GeTe_2_ (FGT) as the spin source and detector and multilayer graphene as the spin transport channel. The nonlocal spin transport signal was found to strongly depend on temperature and disappear at a temperature below the Curie temperature of the FGT flakes, which stemmed from the temperature-dependent ferromagnetism of FGT. The spin injection efficiency was estimated to be about 1%, close to that of conventional nonlocal spin valves with transparent contacts between ferromagnetic electrodes and the graphene channel. In addition, the spin transport signal was found to depend on the direction of the magnetic field and the magnitude of the current, which was due to the strong perpendicular magnetic anisotropy of FGT and the thermal effect, respectively. Our results provide opportunities to extend the applications of van der Waals heterostructures in spintronic devices.

## 1 Introduction

Devices based on spintronics and two-dimensional (2D) van der Waals (vdWs) materials are potential candidates for developing next-generation integrated circuits.^1−3^ On the one hand, spin-based devices, such as spin field-effect transistors,^4^ spintronic memory,^5^ and all-spin logic devices,^6^ provide low-energy budgets. On the other hand, 2D materials offer new opportunities to fabricate novel devices.^7−10^ The combination of spintronics and 2D materials started with fabricating local^11^ and nonlocal^12^ spin valves (NLSVs) with graphene as the transport channel. Then, it quickly expanded to devices to manipulate spins,^13−16^ enhance spin transport,^17−21^ and increase the spin injection efficiency.^22^ With the help of h-BN to maintain the material and structure integrity, the spin relaxation length has been increased to 30.5 μm at room temperature in graphene.^17^ Multilayer graphene has also been employed to transport spin current since its outer layers can screen scattering potentials.^23,24^ The study of 2D spintronics entered a new era with the discovery of 2D ferromagnets,^25−29^ making devices composed of all-2D materials possible. Fe_3_GeTe_2_ (FGT) was discovered as a vdWs ferromagnetic metal with a high Curie temperature of up to 220 K (bulk state) and strong perpendicular magnetic anisotropy.^27,30^ Moreover, the resistivity of FGT is more similar to that of multilayer graphene than those of the traditional 3d transition ferromagnetic metals,^27^ and the reduced conductivity mismatch may enhance the spin injection efficiency.^31,32^ Therefore, FGT can be used to inject and detect spin currents in a multilayer graphene channel. In addition, FGT has been utilized to build vertical magnetic tunnel junctions with all-2D vdWs materials.^33−37^ For example, the FGT/BN/FGT heterostructures showed a tunneling magnetoresistance as high as 160% due to the high-quality interfaces between vdWs materials.^35^ Recently, 2D vdWs ferromagnetic metals, Fe_5_GeTe_2_ and Fe_3_GaTe_2_, with the Curie temperature near^38^ or above^39^ room temperature have been discovered. The tunnel magnetoresistance of up to 85% has been realized in Fe_3_GaTe_2_/WSe_2_/Fe_3_GaTe_2_ heterostructures at room temperature.^40^ In addition, 2D Fe_5_GeTe_2_ and cobalt have been employed to fabricate NLSVs at room temperature.^41^ However, whether an all-2D vdWs NLSV can be fabricated and more importantly whether their performance can be improved by the high-quality interfaces have not been investigated yet.

In this study, NLSVs with two FGT electrodes and a multilayer graphene channel were fabricated using a deterministic transfer method.^42^ The charge current was injected from one FGT flake, and the spin transport signal was detected using the other FGT flake. The nonlocal spin transport signal was observed, and the spin injection efficiency was estimated to be about 1%. The dependence of the spin transport on the direction of the magnetic field and the magnitude of the current was also investigated.

## 2 Results and Discussion

**Figure 1:**
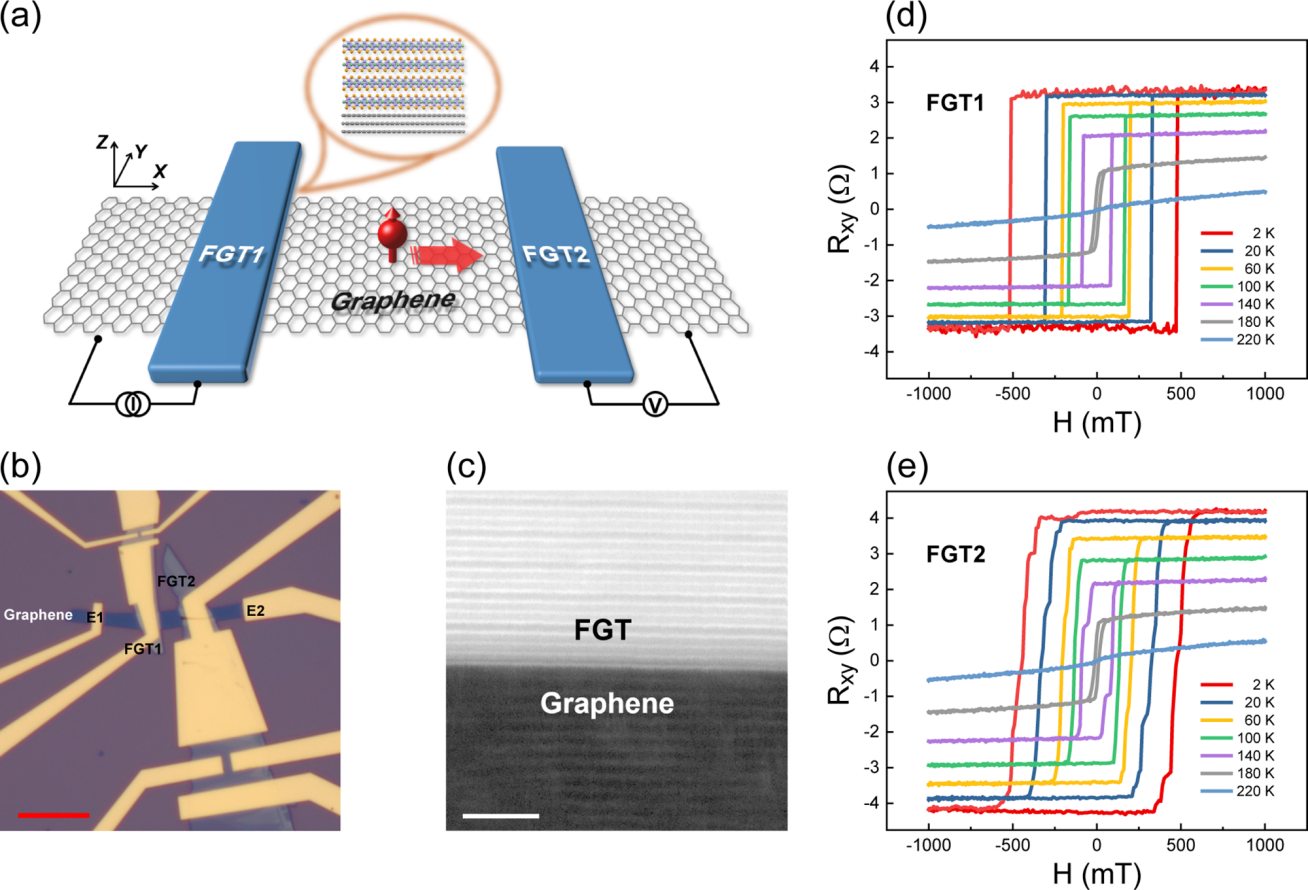
(a) Schematic of the fabricated NLSV device. (b) Optical image of a typical NLSV, comprising a multilayer graphene channel and two FGT electrodes. The scale bar is 10 μm. (c) Cross-sectional STEM image of a typical graphene/FGT heterostructure. The scale bar is 2 nm. (d) Temperature-dependent hysteresis loop of *R_xy_* of FGT1 in (b). (e) Temperature-dependent hysteresis loop of *R_xy_* of FGT2 in (b).

Figure 1a,b shows the schematic and the optical image of our NLSVs, respectively. The device fabrication details can be found in the Experimental Section. The NLSV comprised two FGT flakes (FGT1 and FGT2) and a graphene strip, which acted as the spin injector, the spin detector, and the spin transport channel, respectively. Furthermore, the two FGT flakes differed in thickness and geometry, so their coercive fields should be different,^30,35^ and thus their magnetic moments could be manipulated separately. The atomic force microscopy (AFM) images in Figure S1 indicate that the thicknesses of the graphene channel, FGT1, and FGT2 were 10 nm (∼29 layers), 23 nm (∼28 layers), and 16 nm (∼20 layers), respectively. Two Cr/Au (10 nm/70 nm) electrodes (E1 and E2) were fabricated using electron-beam lithography and subsequent electron-beam evaporation. The distance between the injector (FGT1) and the detector (FGT2) was about 3 μm. Figure 1c shows the cross-section image of a graphene/FGT heterostructure obtained by scanning transmission electron microscopy (STEM). A clear and sharp interface can be observed between graphene and the FGT layer, indicating the high quality of the heterostructure. Furthermore, the contact resistances of the graphene/FGT heterostructure were both lower than 200 Ω, indicating that the contacts were transparent. To characterize the magnetic properties of FGT1 and FGT2, we fabricated a Hall bar device on FGT1 and FGT2 electrodes, respectively (Figure 1b). It is worth mentioning that the electrodes on FGT1 and FGT2, right above the graphene channel, were fabricated to cover the majority of the FGT flakes so as to decrease the spins absorbed by the FGT edge and reduce the current density that may damage the FGT flake (Note S1 andFigure S2). The Hall resistances (*R_xy_*) as a function of the applied magnetic field at different temperatures are shown in Figure 1d,e, respectively. The magnetic field was applied perpendicular to the sample plane, i.e., along the magnetic easy axis of FGT.^30^ Note that the magnetic field in this study was always applied perpendicular to the sample plane unless otherwise specified. The nearly square hysteresis loops of *R_xy_* of FGT1 and FGT2 indicate that the reversal of the magnetic moment in FGT1 or FGT2 was realized through the coherent rotation of a single domain. The kinks observed near the coercive fields in the hysteresis loops of *R_xy_* of FGT2 might relate to defects or local thickness variations in FGT2, which changed the coercive field locally. The hysteresis loops narrowed with the increase in temperature (up to 200 K) (hysteresis loops measured at more temperatures are shown in Figure S3), indicating that the Curie temperatures of FGT1 and FGT2 were around 200 K, which agreed with the previous reports.^30^

**Figure 2:**
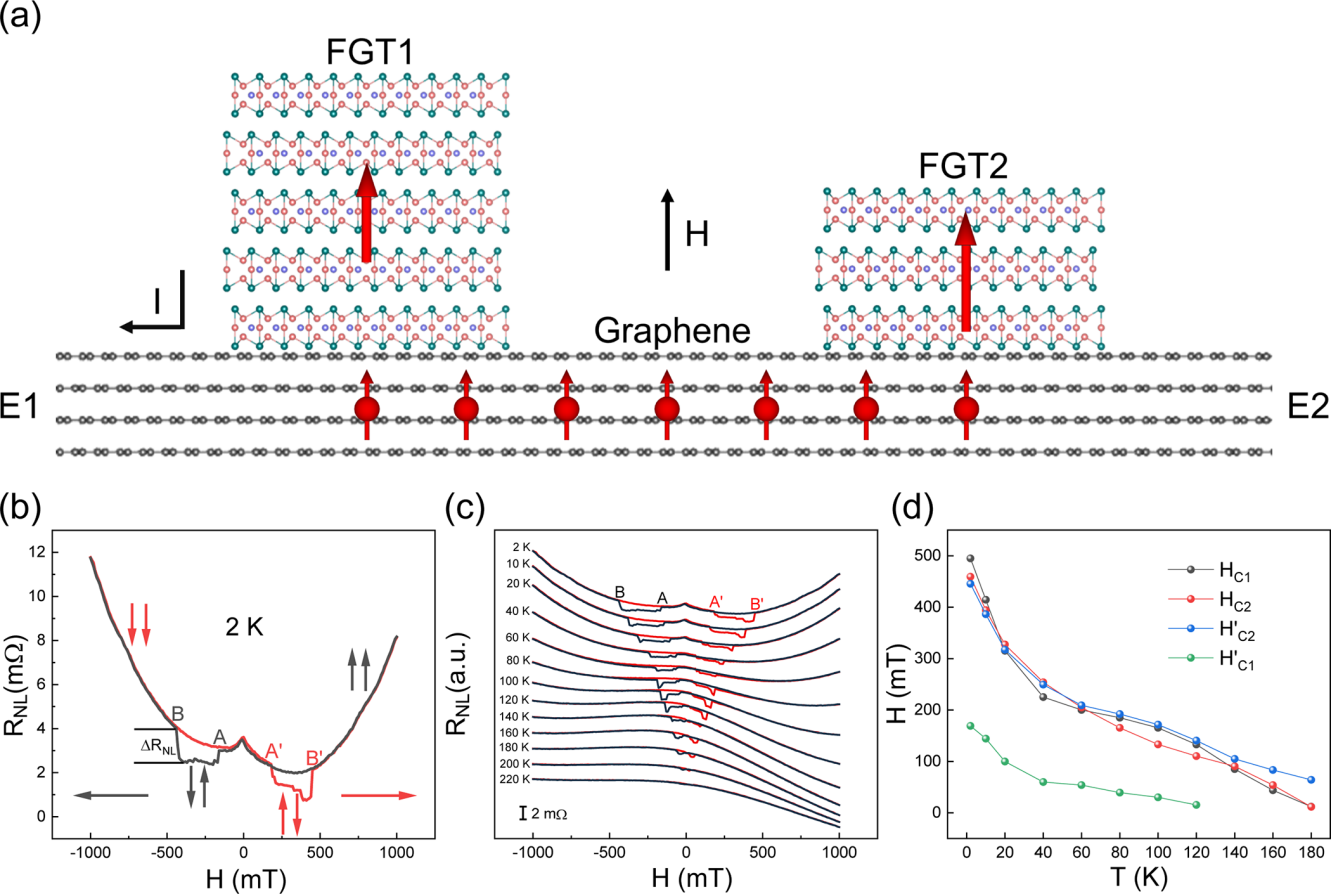
(a) Schematic of the experimental setup for spin transport measurements. The red arrows indicate the magnetization directions of the FGT flakes. The red arrows with spheres represent spin diffusion. (b) Nonlocal resistance of the device in Figure 1b as a function of the magnetic field. (c) Nonlocal resistance of the device in Figure 1b as a function of the magnetic field at various temperatures. The horizontal and vertical arrows in (b) represent the sweeping directions of the magnetic field and the magnetic moments of FGT1 and FGT2, respectively. (d) Coercive fields (H_C1_ and H_C2_) extracted from Figure 1d,e, respectively, and “modified” coercive fields (H'_C2_=(H_B_ + H_B'_)/2 and H'_C1_=(H_A_ + H_A'_)/2) extracted from (c), as a function of temperature (H_A_, H_A'_, H_B_, and H_B'_ are the corresponding magnetic fields for A, A', B and B', respectively).

As shown in Figure 2a, when a perpendicular magnetic field is applied and a current (*I*) is injected from FGT1 to E1, spins accumulate underneath FGT1 and diffuse to both sides of the graphene channel.^24^ When the spin current reaches FGT2, a voltage drop *V*_NL_ develops between FGT2 and E2; the nonlocal resistance *R*_NL_ can be defined as *R*_NL_ = *V*_NL_/*I*.^24^ Different from conventional NLSVs,^12,43^ the magnetization in this study was perpendicular to the sample plane.^27,30^ Due to the different coercivities in FGT1 and FGT2, when the magnetic field was swept between −1000 and 1000 mT, the magnetic configuration between FGT1 and FGT2 changed from parallel to antiparallel and then parallel again, as indicated by the vertical arrows in Figure 2b. Since FGT2 detected the electrochemical potential of the spins diffused from FGT1, a sudden change of *V*_NL_ and *R*_NL_, i.e., Δ*V*_NL_ and Δ*R*_NL_, occurred during the field sweep,^44^ as shown in Figure 2b,c. Several characteristics observed in Figure 2b,c starkly differed from those of conventional NLSVs. First, the background resistances varied with the magnetic field, unlike the constant background in conventional NLSVs.^12^ This happened because part of the injected charge current “leaked” to the detector and induced magnetoresistance and Hall resistance that varied with the perpendicular magnetic field. Meanwhile, the magnetoresistance and the Hall resistance were superimposed on the spin signal. Second, Δ*R*_NL_ was surprisingly weak even when the applied current reached 0.8 mA (Figure 2b). Three factors might lead to this phenomenon: (1) the channel between the injector and the detector was as long as 3 μm (Figure 1b), which weakened the spin signal. This can be verified by the NLSV with a 0.7 μm long channel (Figure S4), whose spin signal was much enhanced already with a current of only 0.3 mA, as shown in Figure S5a. (2) Part of the spins was absorbed by FGT, similar to that observed previously;^45^ and (3) there was still a conductivity mismatch between FGT and graphene, so the backflow of spin current could not be avoided. Third, the magnetic fields where the nonlocal resistances abruptly decreased (A and A′ in Figure 2b) were much lower than the coercive fields of FGT1 (Figure 1d) and FGT2 (Figure 1e) at 2 K, whereas the magnetic fields where the nonlocal resistances abruptly increased (B and B′ in Figure 2b) were close to them, which was in contrast to the scenario in the case of the vertical spin valve.^35^ This arose from the large current-induced temperature rise in the device,^46^ which decreased the coercive field (Figure 1d,e).^27,30^ Since the large current was injected from FGT1 to E1, the coercive field of FGT1 was reduced more. Therefore, the magnetic fields at points A and A′ in Figure 2b correspond to the “modified” coercive field of FGT1, and the magnetic fields at points B and B′ correspond to the “modified” coercive field of FGT2. The relationships between the nonlocal spin signal and temperature are presented in Figures 2c and S5b. With increasing temperature, the magnetic fields where the nonlocal resistances changed abruptly all decreased, following the relationships between the coercive field and temperature (Figure 1d,e), as shown in Figure 2d. It is worth noting that the nonlocal spin signal of the device shown in Figure S4a vanished at 160 K (Figure S5b), much lower than the Curie temperature,^30^ indicating that a more significant thermal effect was induced in the device. To verify the reliability of our results, the injector and the detector were switched with each other, and then the same experiments were performed. We observed similar phenomena, except for different background signals, as shown in Figure S6. This implied that, unlike the previous study,^12^ the nonlocal spin signal in our experiment had strong temperature dependence, stemming from the temperature-dependent ferromagnetism of FGT.^27,30^

**Figure 3:**
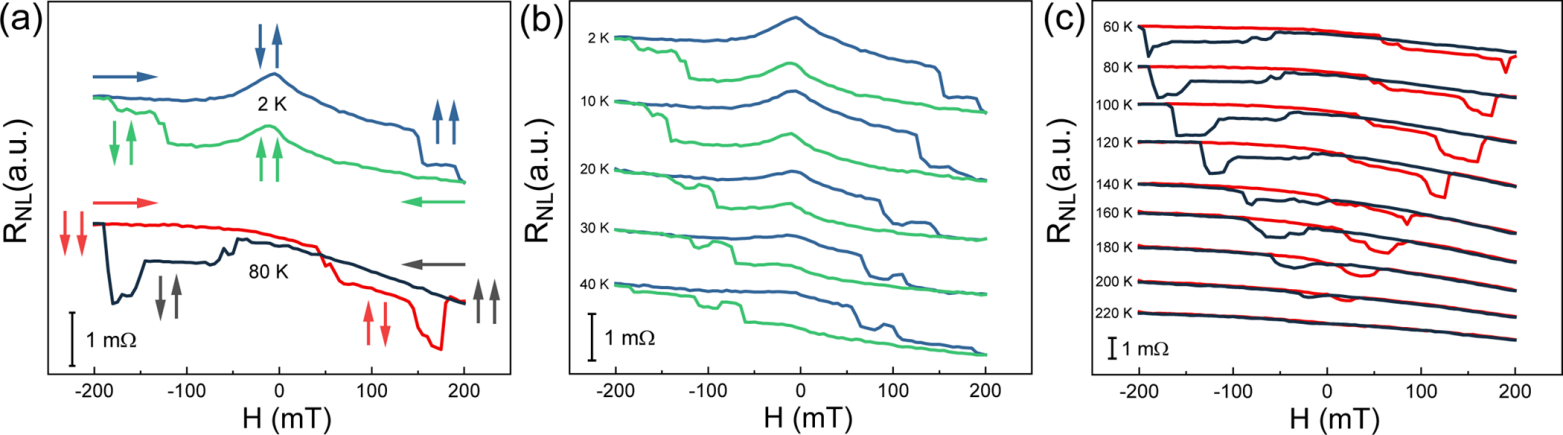
(a) Nonlocal resistance of the device in Figure 1b as a function of the magnetic field at different temperatures. (b) Below 60 K, only the magnetic moment of FGT1 was reversed. (c) At and above 60 K, the magnetic moments of both FGT1 and FGT2 were reversed. The horizontal and vertical arrows in (a) indicate the sweeping directions of the magnetic field and the magnetic moments of FGT1 and FGT2, respectively.

To get more insights into the NLSVs, we swept the magnetic field in a narrower range from −200 to 200 mT (Figure 3). Thus, the field could only reverse the magnetic moment of FGT1 at 2 K, forming a “minor loop” (Figure 3a),^12^ whereas at a higher temperature (80 K), the coercive fields of both FGT1 and FGT2 decreased below 200 mT, and the “normal” nonlocal resistance similar to that in Figure 2b appeared again (Figure 3a). Moreover, in the low- and high-temperature cases in Figure 3a, several “steps” appeared in the nonlocal resistances. This could be attributed to the switching of local magnetic moments in FGT2 since the coercive field of FGT2 might be modified locally by defects or local thickness variations, as described before on the hysteresis loops of *R_xy_* of FGT2. Increasing the temperature from 2 to 40 K, the loop narrowed monotonically (Figure 3b), implying a decreased coercive field. When the temperature rose to 60 K, the curve transformed from one loop (Figure 3b) to two loops (Figure 3c), implying that the magnetic moments of both FGT1 and FGT2 were switched completely.^12^

To extract more information from the NLSVs, we also performed spin precession experiments. We applied a magnetic field in the *z*-direction to preset FGT1 and FGT2 in a parallel or antiparallel magnetic configuration. Subsequently, an in-plane magnetic field was applied parallel to the graphene channel in the *x*-direction (Figure S7) to observe the Hanle effect.^43^ However, even with the magnetic field and the current reaching 3000 mT and 0.8 mA, respectively, no Hanle precession signal was detected. We argue that the absence of the Hanle effect could be attributed to the wide FGT electrodes and spin absorption by the FGT electrodes. The widths of the FGT electrodes were either comparable to (Figure 1b) or larger than (Figure S4a) the channel length. Hence, the injected spins, coming from the different portions of FGT, did not have the same phase and counteracted each other, weakening the Hanle precession signal. In addition, spin absorption by the FGT electrodes shortened the effective spin relaxation time, further making the Hanle precession signal difficult to be detected.

The spin injection efficiency of an NLSV can be estimated using , where Δ*R*_NL_ is the change of the nonlocal resistance, *σ*_G_ is the conductivity of the graphene channel, *P*_J_ is the spin injection efficiency, *λ*_G_ is the spin relaxation length of the graphene channel, and *W* and *L* are the width and the length of the graphene channel between the injector and the detector, respectively.^22^ Since the Hanle effect was absent in our experiment, the spin relaxation length could not be determined. Hence, we assumed a typical value of *λ*_G_ (∼1.5 μm) for multilayer graphene by referring to our previous study.^47^ Moreover, Δ*R*_NL_ was ∼1.5 mΩ (Figure 2b), *σ*_G_ was measured to be 6.1 mS, *W* was ∼2.5 μm, and *L* was ∼3 μm, so *P*_J_ was calculated to be about 1%, close to the result achieved from the NLSV with transparent contacts.^48^ Such a low injection efficiency could be attributed to two factors: first, there was still a conductivity mismatch between FGT and multilayer graphene, and thus the backflow of spins could not be avoided completely. Second, spin absorption by FGT further lowered the injection efficiency.

**Figure 4:**
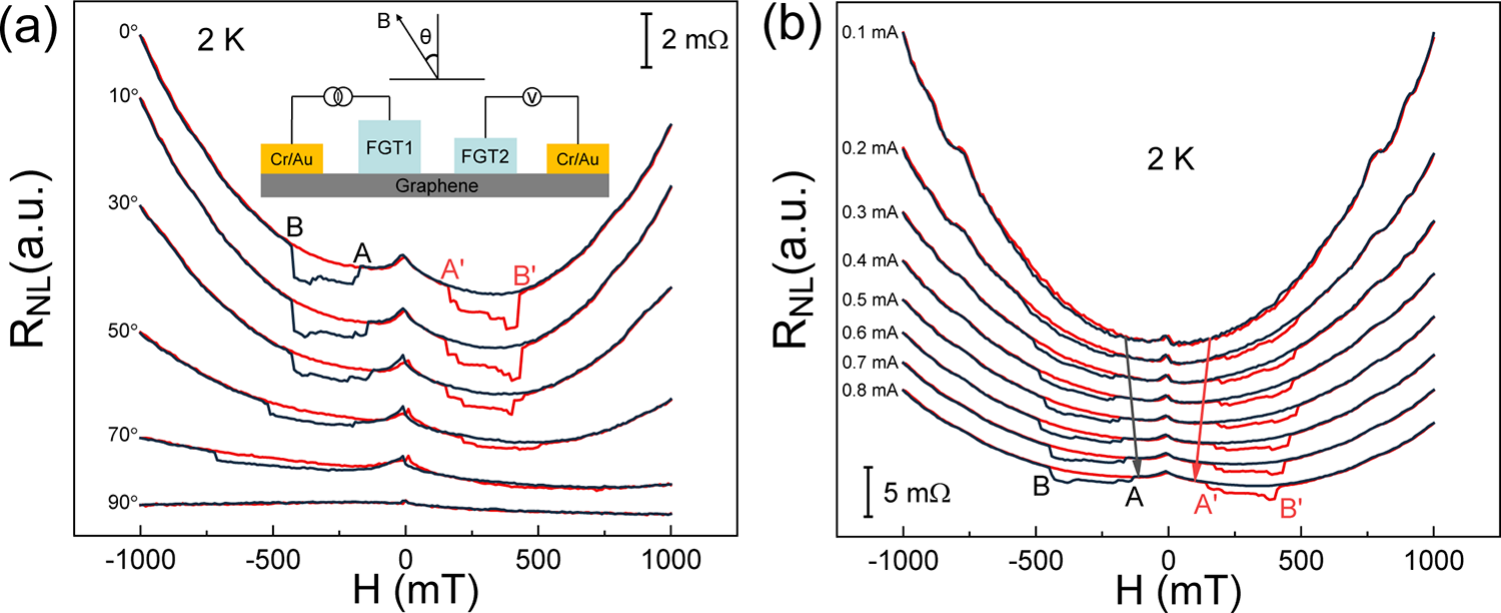
Nonlocal resistance of the device in Figure 1b as a function of (a) magnetic field along different directions and (b) magnetic field injecting currents of different magnitudes. The inset in (a) defines the direction of the magnetic field.

We also investigated the factors that might influence spin transport in our NLSVs. First, we varied the direction of the applied magnetic field (indicated by the angle θ in Figure 4a), and the results at 2 K are presented in Figure 4a. The spin transport signal was barely changed when the angle was less than or equal to 30°. When the angle reached 50°, the magnetic fields for points B and B′ increased suddenly and almost doubled when the angle reached 70°. Finally, the signal disappeared completely at an angle of 90°. This happened because the magnetic easy axis of FGT is perpendicular to the sample plane. Thus, only the component of the magnetic field along the magnetic easy axis contributed to the flip of the magnetic moment. This behavior was also consistent with the relationship between the coercive field of FGT and the direction of the applied magnetic field.^30^ Second, we altered the current from 0.1 to 0.8 mA with a 0.1 mA step and observed the evolution of the nonlocal resistance at 2 K, as shown in Figure 4b. When the current was 0.1 mA, the nonlocal signal was too weak to be observed. After increasing the current to 0.2 mA, a weak nonlocal signal appeared. On further increasing the current, the signal was enhanced, and the magnetic fields for points A and A′ and points B and B′ moved toward zero, indicating the decrease of the coercive fields of FGT1 and FGT2 due to the thermal effect.

## 3 Conclusions

In conclusion, we successfully fabricated NLSVs using all-2D vdWs materials, and we found that spin transport in these NLSVs strongly depended on temperature. We analyzed the causes for the unusual spin transport signal and the absence of the Hanle effect in our NLSVs. In addition, we also investigated the influences of the direction of the magnetic field and the magnitude of the current on the spin transport of the NLSVs. Our study deepens the understanding of NLSVs made of all-2D vdWs materials.

## 4 Experimental Section

### 4.1 Device Fabrication

NLSVs were fabricated in a glove box in an Ar atmosphere (O_2_ < 0.1 ppm, H_2_O < 0.1 ppm). Graphene flakes were first exfoliated from a natural graphite crystal (HQ graphene) onto a Si/SiO_2_ (500 μm/300 nm) substrate. Then, a multilayer graphene strip with the appropriate size was located by an optical microscope (Axio Scope.A1 MAT, Carl Zeiss). Subsequently, FGT flakes were exfoliated from an FGT bulk (HQ graphene) onto polydimethylsiloxane (PDMS) films, and two FGT flakes with different thicknesses on two PDMS films were identified under the optical microscope via their different optical contrasts, respectively. Afterward, the two FGT flakes were transferred and stacked onto the chosen graphene strip with the help of an accurate transfer platform (E1-M, Metatest). After that, the heterostructure was spin-coated with poly(methyl methacrylate) (PMMA) solution and moved outside of the glove box for further fabrication. Finally, Cr/Au (10 nm/70 nm) electrodes were fabricated by electron-beam lithography, followed by electron-beam evaporation. After the fabrication, the samples were quickly moved into a high-vacuum chamber for thermal annealing (∼2 × 10^–5^ Pa, 200 ^°^C, 4 h), which improved the coupling between FGT and graphene.

### 4.2 Characterization

NLSV devices were delivered into a focused ion beam scanning electron microscope (Helios G4, FEI) to fabricate STEM samples. Then, a monochromated Cs-corrected high-resolution STEM instrument (Titan 80-300, FEI) was employed to characterize the interfacial structure of the graphene/FGT heterostructures. The thicknesses of the samples were measured by an AFM instrument (MFP-3D, Asylum Research) using AC mode after performing electronic measurements.

### 4.3 Electrical Transport Measurements

NLSV devices were moved into a physical property measurement system (DynaCool PPMS, Quantum Design) for measurements. During the measurements, an alternating current at 13 Hz was applied by a Keithley 6221 current source, and the voltage was measured by an SR830 lock-in amplifier. A current of 1 μA was applied to the devices during the anomalous Hall-effect measurements to avoid the thermal effect.

## Supporting Information Available

Optical and AFM images of device 1, the reason for increasing the area of the cap layer on FGT, comparison between the conventional NLSV and our NLSV, additional hysteresis loops of *R_xy_* of FGT1 and FGT2 in device 1 at different temperatures, optical and AFM images of device 2, nonlocal resistance of device 2 as a function of the magnetic field at various temperatures, nonlocal resistance of devices 1 and 2 as a function of the magnetic field switching the injector and the detector at various temperatures, and schematic setup for the Hanle-effect measurements (PDF)
