# Supplementary material for: Nonlocal Spin Valves Based on Graphene/Fe3GeTe2 van der Waals Heterostructures
Source: ACS Appl Mater Interfaces. 2023 Feb 8;15(7):9649–55. doi: 10.1021/acsami.2c21918 (PMC9951179; doi:10.1021/acsami.2c21918)
Supplement: Supplementary file 1 — am2c21918_si_001.pdf [file am2c21918_si_001.pdf]

Supporting Information

Nonlocal Spin Valves Based on Graphene/Fe_3GeTe_2 van der Waals Heterostructures

Xin He,[*,†] Chenhui Zhang,[†] Dongxing Zheng,[†] Peng Li,[‡] John Q. Xiao,[§] and Xixiang Zhang[*,†]

[†]Physical Science and Engineering Division, King Abdullah University of Science and Technology (KAUST), Thuwal 23955-6900, Saudi Arabia

[‡] State Key Laboratory of Electronic Thin Film and Integrated Devices, University of Electronic Science and Technology of China, Chengdu 610054, China

[§]Department of Physics and Astronomy, University of Delaware, Newark, Delaware 19716, United States

[*]E-mail: xin.he@kaust.edu.sa.

[*]E-mail: xixiang.zhang@kaust.edu.sa.

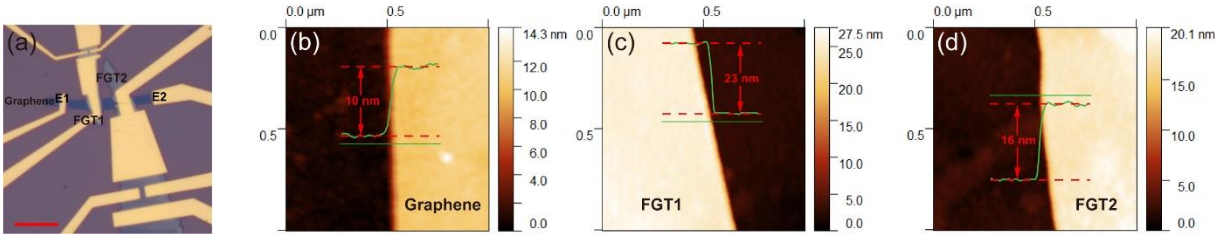

Figure S1. (a) Optical image of a typical nonlocal spin valve (NLSV) (device 1) used in our experiment. The scale bar is 10 μm. (b), (c) and (d) show the AFM images of the graphene channel, FGT1, and FGT2 in (a), respectively.

Note S1. The reason for increasing the area of the cap layer on FGT

In a conventional NLSV (Figure S2a), the conductivity of the ferromagnetic electrode (e.g., Co) is high, and the areas of the cap layer (e.g., Au) and the ferromagnetic electrode in an NLSV are always identical because the metals are evaporated on the same pattern one after another. Thus, the current density can be seen as uniform in the ferromagnetic electrode. While in our NLSV (Figure S2b), the conductivity of FGT is much lower than that of 3d transition ferromagnetic metal. If the cap layer (marked by C in Figure S2b) and FGT flake differ widely in area, the current density right below the cap layer will be much higher than that of the uncovered areas. As shown in Figure S2b, spins need to diffuse to another FGT flake to be detected, thus some of them would be absorbed by the FGT edge (marked by A in Figure S2b) that creates a smaller spin current or even no spin current due to the much lower charge current density there. Therefore, we should widen the cap layer to decrease the spins absorbed. Moreover, the current applied in our experiment was large, we needed to increase the area of the cap layer so that the local current density would not be too high to damage the FGT layer.

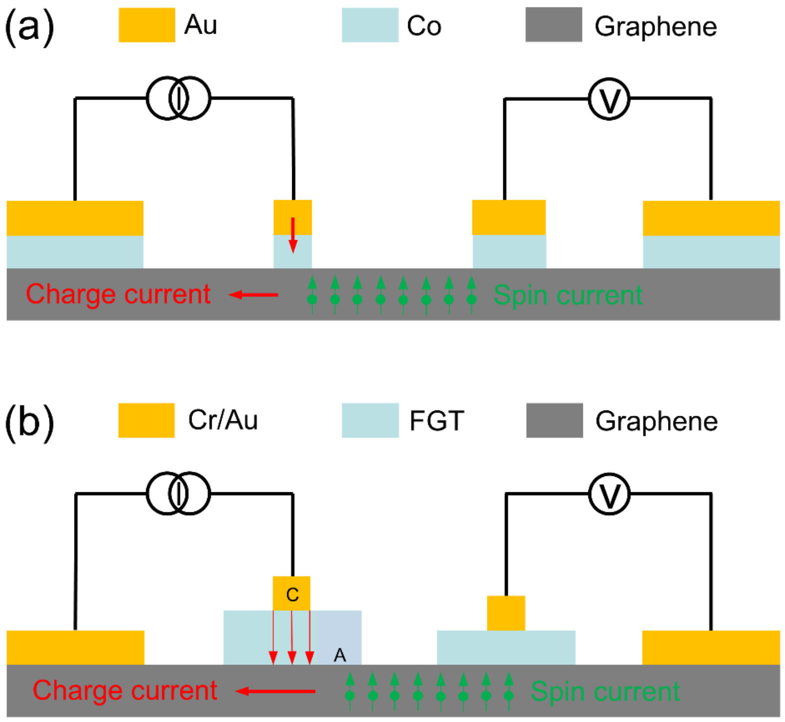

Figure S2. Comparison between the conventional NLSV (a) and our NLSV (b). The red arrows represent the charge current while the green arrows with disks represent the spin current.

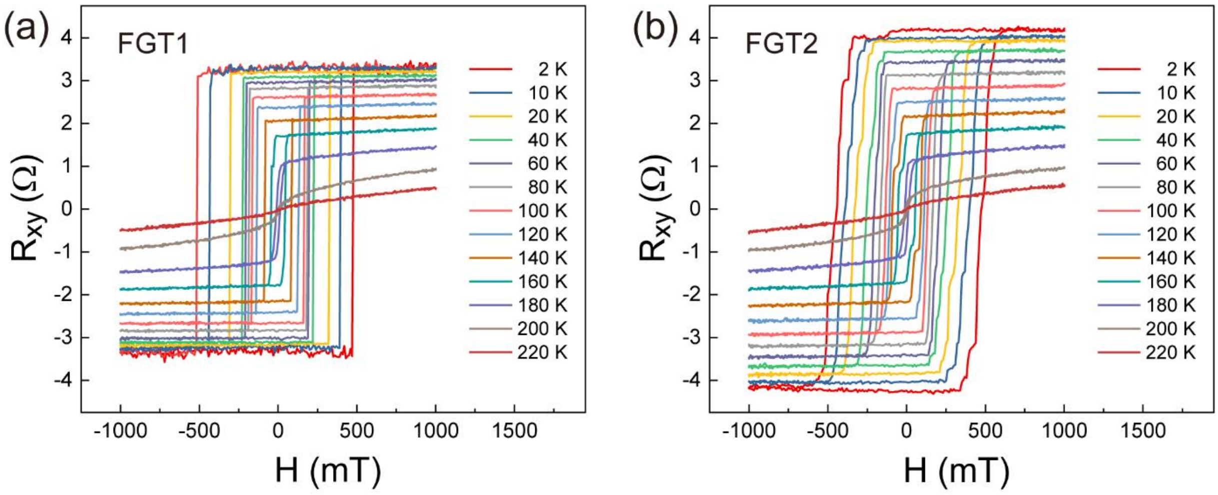

Figure S3. (a) Temperature-dependent hysteresis loop of R_{xy} of FGT1 in Figure 1b. (b) Temperature-dependent hysteresis loop of R_{xy} of FGT2 in Figure 1b.

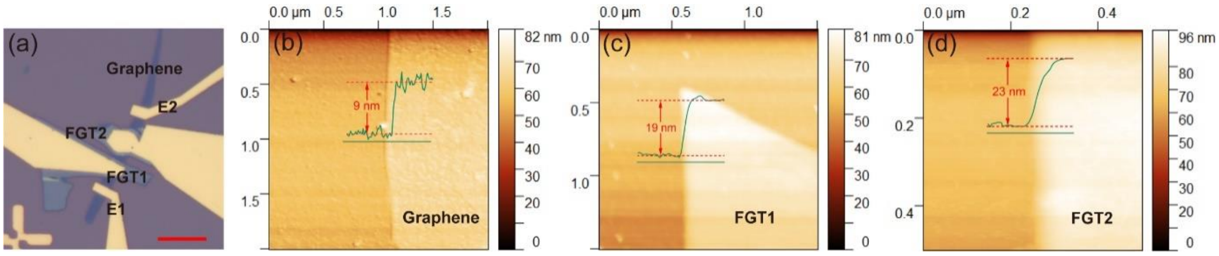

Figure S4. (a) Optical image of a typical NLSV (device 2) used in our experiment. The scale bar is 10 μm. (b), (c) and (d) show the AFM images of the graphene channel, FGT1, and FGT2 in (a), respectively.

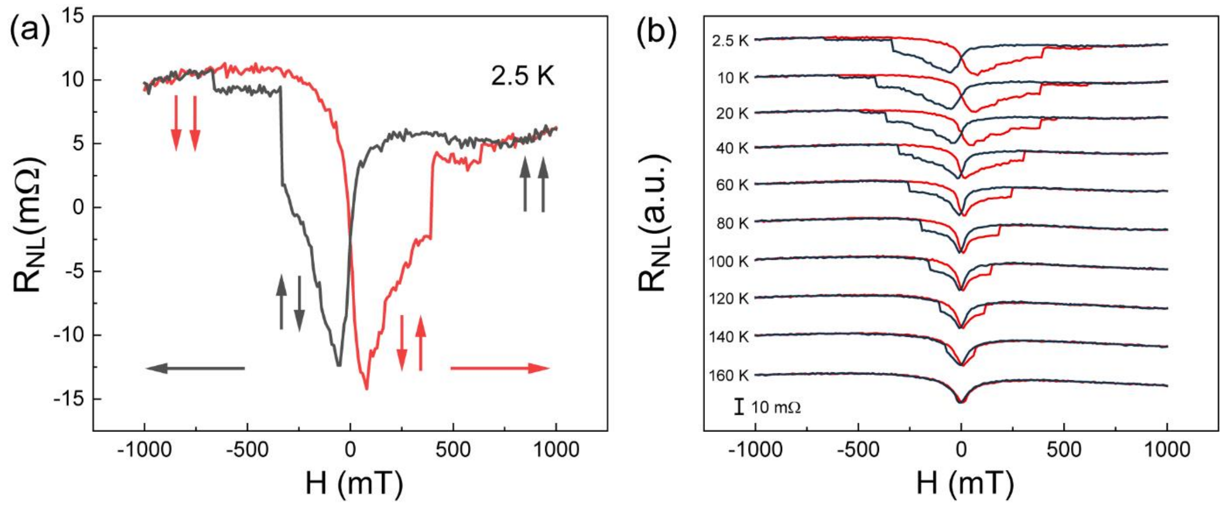

Figure S5. (a) Nonlocal resistances of the devices in Figure S4a (device 2) as a function of the magnetic field. (b) Nonlocal resistances of the devices in Figure S4a (device 2) as a function of the magnetic field at various temperatures. The horizontal and the vertical arrows in (a) represent the sweeping directions of the magnetic field and the magnetic moments of FGT1 and FGT2, respectively.

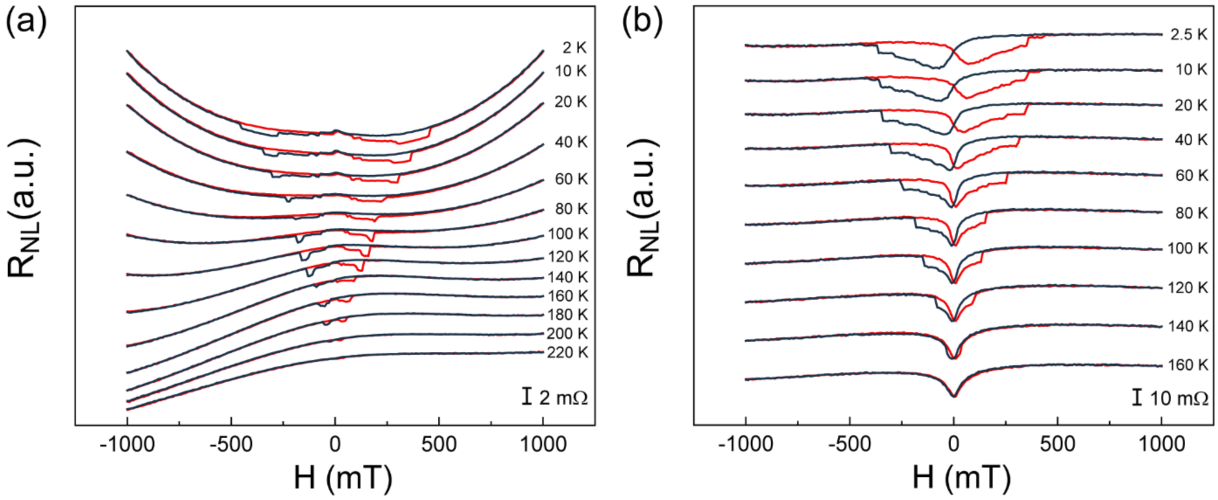

Figure S6. (a) Nonlocal resistance of the device in Figure 1b (device 1) as a function of the magnetic field at various temperatures, switching the injector and the detector. (b) Nonlocal resistance of the device in Figure S4a (device 2) as a function of the magnetic field at various temperatures, switching the injector and the detector.

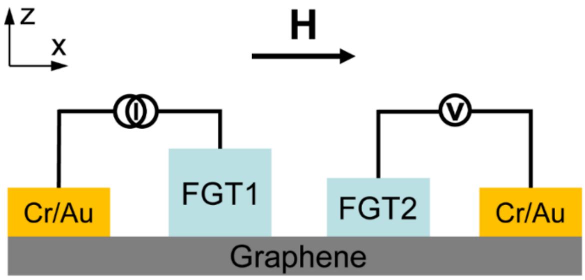

Figure S7. Schematic setup for the Hanle-effect measurements.
